# The potential of rectal swabs to differentiate simple and complex appendicitis in children with a microbiota-based test

**DOI:** 10.1007/s00431-022-04627-0

**Published:** 2022-10-04

**Authors:** Sarah-May M. L. The, Tim G. J. de Meij, Andries E. Budding, Roel Bakx, Johanna H. van der Lee, Linda Poort, Huib A. Cense, Hugo A. Heij, L. W. Ernst van Heurn, Ramon R. Gorter

**Affiliations:** 1 Department of Pediatric Surgery, Emma Children’s Hospital, Amsterdam University Medical Centers, location University of Amsterdam, Amsterdam, 1105 AZ The Netherlands; 2 Department of Pediatric Gastroenterology, Emma Children’s Hospital, Amsterdam University Medical Centers, location University of Amsterdam, Amsterdam, 1105 AZ The Netherlands; 3 Inbiome, Amsterdam, 1098 XG The Netherlands; 4 Pediatric Clinical Research Office, Emma Children’s Hospital, Amsterdam University Medical Centers, location University of Amsterdam, Amsterdam, 1105 AZ The Netherlands; 5 Dutch Knowledge Institute, Federation of Medical Specialists, Utrecht, 3528 BL The Netherlands; 6 Department of Surgery, Red Cross Hospital, Beverwijk, 1942 LE The Netherlands; 7 Amsterdam Reproduction and Development Research Institute, Amsterdam, The Netherlands; 8 Amsterdam Gastroenterology and Metabolism Research Insititute, Amsterdam, The Netherlands

**Keywords:** Intestinal microbiota, Rectal swabs, Bacterial infections, Pediatric surgery, IS-pro

## Abstract

Currently, accurate biomarkers differentiating simple (phlegmonous) from complex (gangrenous and/or perforated) appendicitis in children are lacking. However, both types may potentially require different treatment strategies, and the search for diagnostic modalities remains warranted. Previously, we demonstrated a distinct microbiota (both an increased bacterial diversity and abundance) in the appendix of children with complex compared to simple appendicitis. From the same cohort of patients we have collected 35 rectal swabs under general anesthesia prior to appendectomy and microbiota analysis was performed by IS-pro, a 16S-23S rDNA–based clinical microbiota profiling technique. Using the obtained IS-profiles, we performed cluster analyses (UPGMA), comparison of diversity (Shannon Diversity Index) and intensity (abundance in relative fluorescence units) on phylum level, and comparison on species level of bacteria between simple and complex appendicitis. Regarding these analyses, we observed no clear differences between simple and complex appendicitis. However, increased similarity of the microbial composition of the appendix and rectal swab was found within children with complex compared to simple appendicitis. Furthermore, PLS-DA regression analysis provided clear visual differentiation between simple and complex appendicitis, but the diagnostic power was low (highest AUC 0.65).

*Conclusion*: Microbiota analysis of rectal swabs may be viable to differentiate between simple and complex appendicitis prior to surgery as a supervised classification model allowed for discrimination of both types. However, the current diagnostic power was low and further validation studies are needed to assess the value of this method.

**Table:** 

**What is Known:** *•* *Simple and complex appendicitis in children may require different treatment strategies, but accurate preoperative biomarkers are lacking.* • *Clear differentiation can be made between both types in children based upon the microbial composition in the appendix.*	
**What is New:** *• Increased similarity was found between the microbial composition of the appendix and rectal swab within children with complex compared to simple appendicitis.* *•* *Using a supervised classification model rectal swabs may be viable to discriminate between simple and complex appendicitis, but the diagnostic power was low.*

**What is Known:**

*•* *Simple and complex appendicitis in children may require different treatment strategies, but accurate preoperative biomarkers are lacking.*

• *Clear differentiation can be made between both types in children based upon the microbial composition in the appendix.*

**What is New:**

*• Increased similarity was found between the microbial composition of the appendix and rectal swab within children with complex compared to simple appendicitis.*

*•* *Using a supervised classification model rectal swabs may be viable to discriminate between simple and complex appendicitis, but the diagnostic power was low.*

## Introduction

Acute appendicitis is divided into simple and complex appendicitis. As it has become evident that both types require different treatment strategies [1, 2], accurate preoperative differentiation is essential. However, differentiation based on (a combination of) clinical, biochemical, and radiological characteristics remains challenging and novel tools are needed [3].

The microbiota in the appendix has been known to differ between children with simple and complex appendicitis [4, 5]. Jackson et al. reported significant differences in the microbiota of the appendix between children with non-perforated and perforated appendicitis [5]. And, we recently demonstrated a clear differentiation between simple and complex appendicitis based upon the microbial composition in the appendix. This differentiation is characterized by an increase of both diversity and abundance of bacteria in complex appendicitis [4]. If this observed microbial dysbiosis is also reflected in preoperative rectal swabs, it would provide an opportunity for preoperative differentiation and the decision of optimal treatment.

Therefore, the aim of this study was to evaluate whether microbiota analysis of preoperatively collected rectal swabs from children with simple and complex appendicitis could differentiate between both types.

## Materials and methods

### Design and population

This study was part of a larger prospective cohort study, performed between November 2015 and November 2016 (Amsterdam UMC and Red Cross Hospital Beverwijk, The Netherlands). The study was reviewed by the local ethics committee of the Amsterdam UMC (location Vumc) and labelled as non-WMO (act of medical research of human subjects was waived). According to protocol, consent was asked from children (≥ 12 years of age) and parents for both usage of the appendix and the obtainment of a rectal swab separately. Children 0–17 years old with appendicitis, treated with an acute appendectomy, were eligible for inclusion. Children were excluded in case of perioperative suspicion of a malignancy or diagnosis other than appendicitis. An overview of additional methodology and results on the microbial composition of the appendix were previously published [4]. Based upon perioperative and histopathological data, the same blinded classification of children into simple (phlegmonous appendicitis with transmural invasion of neutrophils, in absence of signs of complexity) and complex appendicitis (extensive ulceration, gangrenous or perforated appendix with or without abscess formation or purulent intra-abdominal fluid) was used [4]. The combination of both perioperative and histopathological findings was deliberately chosen to limit the chance of inter-observer variability and sampling error respectively.

Following inclusion, rectal swabs were collected, prior to appendectomy, when children were under general anesthesia. The swabs (Copan swab 520CS01) were obtained according to a standardized procedure, put in a vial with 500 µl of transport fluid and stored directly after surgery at −20 °C. For DNA isolation, the swabs were thawed simultaneously and 1 ml of lysis buffer was added (nucliSENS) [6]. The vial was then shaken, incubated, and centrifuged. Two hundred microliters of the supernatant was suspended in 2-ml lysis buffer (nucliSENS) and incubated. Then, 70 µl of magnetic silica beads was added to each sample. The specific “A” protocol of the machine (easyMAG machine) was used: DNA was eluted in 110-µl extraction buffer (Biomerieux, nuclisens easyMAG extraction buffer 3). Microbiota analysis was then performed by IS-pro, a 16S-23S rDNA–based clinical microbiota profiling technique. This procedure was performed following the manufacturer’s instructions for use (inbiome, Amsterdam) and interpretation of resulting IS-profiles was performed by inbiome Amsterdam, The Netherlands, as previously published [4, 6, 7].

### Microbiota and statistical analysis

Cluster analysis (UPGMA), diversity (Shannon Diversity Index) and intensity analysis (abundance in relative fluorescence units) on phylum level, and comparison of presence on species level were performed in the same manner as was done previously for the appendix [4]. We used TIBCO Spotfire software for visualization of clusters. All statistical analyses were performed with Prism GraphPad, Version 8, using Mann–Whitney *U* test or Fisher’s exact test when appropriate. Statistical significance was determined as *P* < 0.05 and for comparison of species, a Bonferroni correction was performed. In addition, we assessed the correlation between the microbial composition found in the appendix and rectal swab within a child with simple compared to complex appendicitis by an intra-group analysis (similarity expressed as a coefficient, *R* square). Moreover, using Python software version 3.7.9 with Scikit-Learn version 1.0.2, we performed a principal coordinate analysis (PCoA) to visualize dissimilarities on phylum level and partial least squares discriminant analysis (PLS-DA) regression model for the prediction of clinical status (simple and complex appendicitis) [8, 9]. The PLS-DA encompassed all identified OTUs and was performed including a tenfold cross-validation procedure. Results were pooled to compute diagnostic accuracies.

## Results

### General characteristics

Rectal swabs were collected preoperatively from 35 children, 16 with simple and 19 with complex appendicitis. Baseline characteristics were comparable for age, gender, days of abdominal pain, C-reactive protein, and received prophylactic antibiotics. Differences were found for temperature at presentation and leukocyte count (both *P* = 0.014) (Table 1).

**Table 1:** Baseline characteristics

	**All**	**Simple**	**Complex**	***P*-value**
**Number of patients**	35	16	19	
**Age, years**	11 [1–7]	11 [6–17]	12 [1–16]	0.581
**Female sex**	17 (49)	8 (50)	9 (47)	> 0.999
**Abdominal pain, days**	1 [1–8]	1 [1–4]	2 [1–8]	0.161
**Temperature, °C**	37.4 [36.0–40.0]	36.9 [36.3–37.9]	37.6 [36.0–40.0]	0.014
**C-reactive protein, mg/L**	40 [1–262]	25 [1–262]	61 [7–247]	0.057
**Leukocyte count, × 10^9/L**	16.2 [6.0–25.5]	12.3 [6.0–21.0]	18.0 [11.2–25.5]	0.014
**Prophylactic antibiotics**				
Regime 1	28 (80)	14 (88)	14 (74)	0.415
Regime 2	7 (20)	2 (13)	5 (26)	

All data provided as median with [range], except for female sex and use of prophylactic antibiotics which are provided as number with (percentage). Statistical analysis using Mann–Whitney *U* test and Fisher’s exact test when appropriate. Significance defined as *P* < 0.05. Use of prophylactic antibiotics according to local protocol: regime one was metronidazole with cephalosporin; and regime two was amoxicillin with clavulanic acid with or without gentamycin

### Cluster analysis and analysis on phylum level

**Fig. 1:**
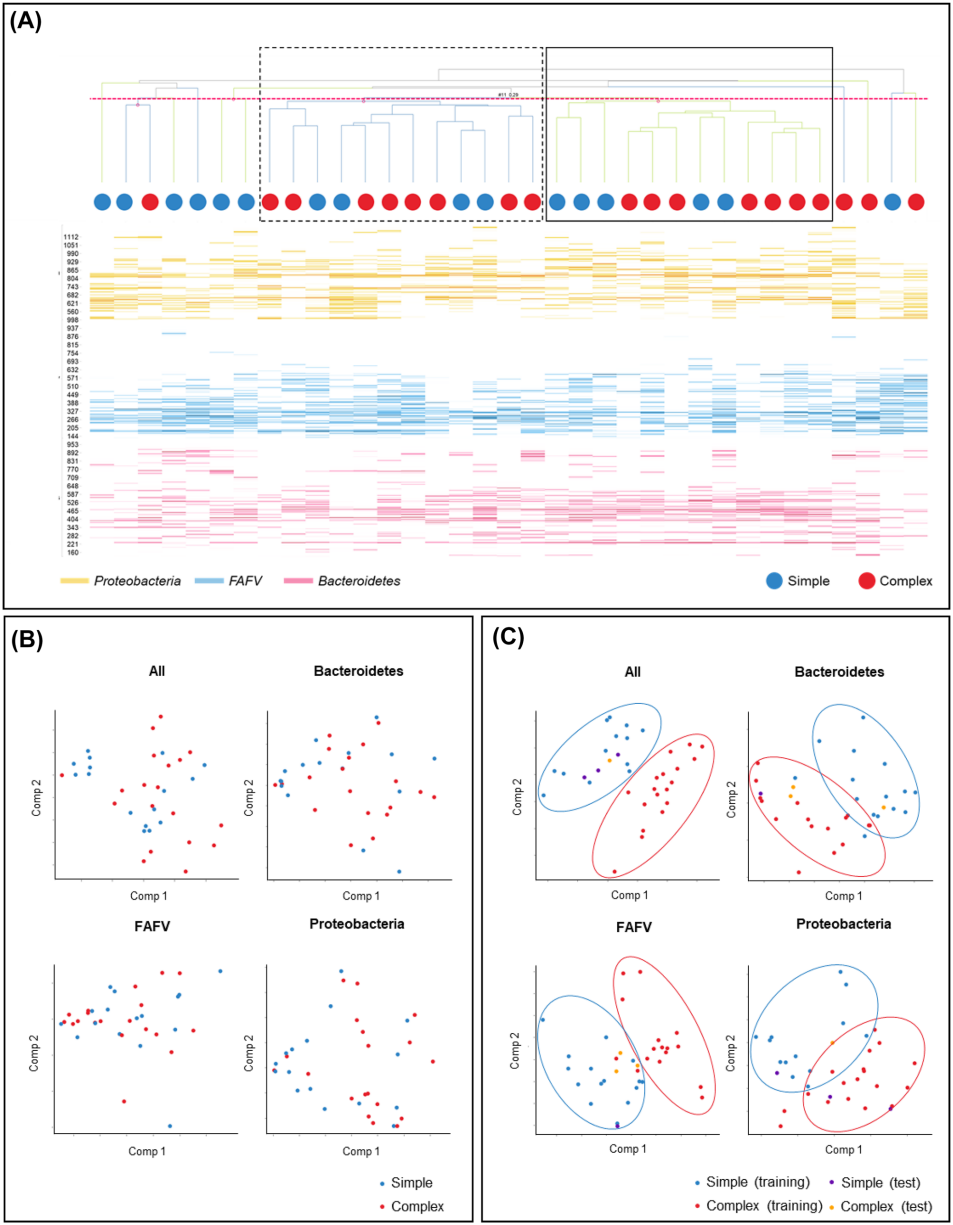
**A** Cluster analysis including the three main phyla combined. **B** PCoA visualization for all phyla combined, and Proteobacteria, Bacteroidetes, and FAFV separately. No clear differentiation could be made. **C** PLS-DA scores with tenfold cross-validation for all phyla combined, and Proteobacteria, Bacteroidetes, and FAFV separately. Clear visual differentiation between children with simple and complex appendicitis. For **A**, **B**, and **C**, patients are represented by color-coded dots

Cluster analysis identified two clusters when all phyla were combined, but showed no association between type of appendicitis (*P* > 0.999) (Fig. 1A). No clusters were identified with analysis of each phylum separately (Bacteroidetes, Proteobacteria, and FAFV). In addition, both diversity and abundance analysis on phylum level showed no significant differences. With pooled intra-group correlation analysis, increased similarities were found between the appendix and rectal swab of children with complex compared to simple appendicitis (median *R*^2^ of simple appendicitis 0.026, [range 0.00–0.27], and median *R*^2^ of complex appendicitis 0.252, [range 0.00–0.38], *P* = 0.0006).

### PCoA and PLS-DA

Figure 1B demonstrates the results of PCoA visualization of dissimilarities. No differentiation could be made for all phyla combined, or one of three phyla separately. By PLS-DA, the profiles of children with simple and complex appendicitis could be visually differentiated as provided in Fig. 1C. The AUC was 0.55 for all phyla combined with sensitivity of 0.49 (95%CI 0.46–0.51) and specificity of 0.46 (95%CI 0.44–0.49); AUC 0.60 for Bacteroidetes with sensitivity of 0.44 (0.42–0.47) and specificity of 0.43 (0.41–0.46); AUC 0.65 for FAFV with sensitivity of 0.46 (0.44–0.48) and specificity of 0.57 (0.55–0.60); and AUC 0.50 for Proteobacteria with sensitivity of 0.57 (0.55–0.59) and specificity of 0.40 (0.37–0.42).

### Analysis on species level

In total, 123 unique species were identified. Differences were found in presence of species for *Klebsiella pneumoniae* (simple 7/16, complex 1/19; *P* = 0.0130), *Cutibacterium acnes* (simple 1/16, complex 10/19; *P* = 0.0041), and *Ruminococcus* spp. (simple 10/16, complex 5/19; *P* = 0.0442), but none of these differences was found to be statistically significant after Bonferroni correction (*P* = 0.351, *P* = 0.1107, and *P* = 0.884 respectively).

## Discussion

Here, we observed no clear differences in microbial composition between simple and complex appendicitis in terms of diversity or intensity on phylum level, with UPGMA cluster analysis, PCoA visualization, or on species level. However, increased similarity was found between the appendix and rectal swab of children with complex compared to simple appendicitis. Furthermore, visual differentiation could be appraised with a supervised classification model between simple and complex appendicitis, but the diagnostic power was low (highest AUC 0.65).

We previously demonstrated a distinct microbiota in the appendix of children with simple and complex appendicitis: Complex appendicitis was associated with increased diversity and abundance on phylum level and increased presence of the species *Allistipes finegoldii*, *Bacteroides fragilis*, *Escherichia coli*, *Parvimonas micra*, and *Sutteralla* spp. [4]. These findings were not appraised in the rectal swabs, and although discrimination between both types was found by PLS-DA, the diagnostic accuracy was low. This lack of accurate differentiation could be due to a relatively small sample size, but the number of samples was comparable to our previous study [4]. More likely, several other factors play a role. A potential explanation is the difference of the samples: the appendix samples consisted of entire sections of the appendix, and included mucosa-associated and possibly invasive bacteria, while the rectal swabs only sample intraluminal bacteria (and potential fecal adherence). And, the appendix harbors local microbiota at the site of inflammation, secured of the fecal stream, while the rectum is anatomically distant [10–12]. We hypothesize that a predominance of this fecal stream of bacteria may subsequently lower the discriminatory potential.

Importantly, the current evaluation by IS-pro analysis takes up to several hours to generate an analyzed microbiota outcome [6, 7]. Ideally, the procurement of results is faster than this as the results are needed to differentiate between patients who can be treated conservatively and those who need immediate surgery. Noteworthy, as it was outside the scope of this study, we did not look into specific costs of sample collection and analysis for individuals separately. In case of implementation of a novel tool, this should be taken into account. And as stated before, for future reference, a larger sample size might still find differences that were too small to observe in this sample set.

## Conclusion

In conclusion, microbiota analysis of rectal swabs may be viable to differentiate between simple and complex appendicitis prior to surgery as a supervised classification model allowed for discrimination of both types. However, the current diagnostic power was low and further validation studies are needed to assess the value of this method.

## Data Availability

Original data are available upon reasonable request.

## References

[1] Gorter RR, The S-MML, Gorter-Stam MAW (2017). Systematic review of nonoperative versus operative treatment of uncomplicated appendicitis. J Pediatr Surg.

[2] Salminen P, Tuominen R, Paajanen H (2018). Five-year follow-up of antibiotic therapy for uncomplicated acute appendicitis in the APPAC randomized clinical trial. JAMA.

[3] Gorter RR, van den Boom AL, Heij HA (2016). A scoring system to predict the severity of appendicitis in children. J Surg Res.

[4] The S-MML, Bakx R, Budding AE (2019). Microbiota of children with complex appendicitis: different composition and diversity of the microbiota in children with complex compared with simple appendicitis. Pediatr Infect Dis J.

[5] Jackson HT, Mongodin EF, Davenport KP (2014). Culture-independent evaluation of the appendix and rectum microbiomes in children with and without appendicitis. PLoS ONE.

[6] Budding AE, Grasman ME, Eck A (2014). Rectal swabs for analysis of the intestinal microbiota. PLoS ONE.

[7] Budding AE, Grasman ME, Lin F (2010). IS-pro: high-throughput molecular fingerprinting of the intestinal microbiota. FASEB J.

[8] Daniels L, Budding AE, de Korte N (2014). Fecal microbiome analysis as a diagnostic test for diverticulitis. Eur J Clin Microbiol Infect Dis.

[9] Hissink Muller P, de Meij TGJ, Westedt M (2017). Disturbance of microbial core species in new-onset juvenile idiopathic arthritis. J Pediatr Infect Dis.

[10] Donaldson GP, Lee SM, Mazmanian SK (2016). Gut biogeography of the bacterial microbiota. Nat Rev Microbiol.

[11] Dieterich W, Schink M, Zopf Y (2018). Microbiota in the gastrointestinal tract. Med Sci.

[12] Bollinger RR, Barbas AS, Bush EL (2007). Biofilms in the large bowel suggest an apparent function of the human vermiform appendix. J Theor Biol.

